# RHBDD1 promotes colorectal cancer metastasis through the Wnt signaling pathway and its downstream target ZEB1

**DOI:** 10.1186/s13046-018-0687-5

**Published:** 2018-02-09

**Authors:** Mengmeng Zhang, Fei Miao, Rong Huang, Wenjie Liu, Yuechao Zhao, Tao Jiao, Yalan Lu, Fan Wu, Xiaojuan Wang, Han Wang, Hong Zhao, Hongge Ju, Shiying Miao, Linfang Wang, Wei Song

**Affiliations:** 1 State Key Laboratory of Medical Molecular Biology, Department of Biochemistry and Molecular Biology, Institute of Basic Medical Sciences, Chinese Academy of Medical Sciences & Peking Union Medical College, Beijing, 100005 China; 2 Department of Abdominal Surgical Oncology, Cancer Hospital & Institute, Chinese Academy of Medical Sciences, Beijing, 100021 China; 3 Department of Pathology, Baotou Medical College, Baotou, 014040 China; 4 Department of Pathology, The First Affiliated Hospital of Baotou Medical College, Baotou, 014010 China

**Keywords:** RHBDD1, Colorectal cancer, Metastasis, Wnt signaling pathway, ZEB1

## Abstract

**Background:**

40–50% of colorectal cancer (CRC) patients develop metastatic disease; the presence of metastasis hinders the effective treatment of cancer through surgery, chemotherapy and radiotherapy, which makes 5-year survival rate extremely low; therefore, studying CRC metastasis is crucial for disease therapy. In the present study, we investigated the role of rhomboid domain containing 1 (RHBDD1) in tumor metastasis of CRC.

**Methods:**

The expression of RHBDD1 was analyzed in 539 colorectal tumor tissues for its correlation with lymphatic metastasis and distal metastasis. Transwell assay in vitro and pleural metastasis analysis in vivo were performed to determine the functions of RHBDD1 during CRC cells metastasis. RNA-seq analysis, TOP/FOP flash reporter assay, western blot and transwell assay were performed to investigate the underlying mechanism for the function of RHBDD1 on Wnt signaling pathway. Bioinformatics analysis was conducted to investigate epithelial-mesenchymal transition (EMT) and stemness in HCT-116 cells. Tissue microarray analysis, Q-PCR and western blot were performed to determine the correlation of RHBDD1 and Zinc Finger E-Box Binding Homeobox 1 (ZEB1).

**Results:**

In this study, we found that RHBDD1 expression was positively correlated with lymphatic metastasis and distal metastasis in 539 colorectal tumor tissues. RHBDD1 expression can promote CRC cells metastasis in vitro and in vivo. RNA-Seq analysis showed that the Wnt signaling pathway played a key role in this metastatic regulation. RHBDD1 mainly regulated ser552 and ser675 phosphorylation of β-catenin to activate the Wnt signaling pathway. Rescuing ser552 and ser675 phosphorylation of β-catenin resulted in the recovery of signaling pathway activity, migration, and invasion in CRC cells. RHBDD1 promoted EMT and a stem-like phenotype of CRC cells. RHBDD1 regulated the Wnt/β-catenin target gene ZEB1, a potent EMT activator, at the RNA and protein levels. Clinically, RHBDD1 expression was positively correlated with ZEB1 at the protein level in 71 colon tumor tissues.

**Conclusions:**

Our findings therefore indicated that RHBDD1 can promote CRC metastasis through the Wnt signaling pathway and ZEB1. RHBDD1 may become a new therapeutic target or clinical biomarker for metastatic CRC.

**Electronic supplementary material:**

The online version of this article (10.1186/s13046-018-0687-5) contains supplementary material, which is available to authorized users.

## Background

Cancer metastasis accounts for 90% of deaths among tumor patients [1]. Colorectal cancer (CRC) mainly metastasizes to the lung and liver [2], and 40–50% of CRC patients develop metastatic disease [3]. The presence of metastasis at diagnosis or metastatic recurrence hinders the effective treatment of cancer, making these patients unsuited for chemotherapy and radiotherapy. The 5-year survival rate in patients with distant tumor spread is slightly greater than 10% [4].

Approximately 90% of CRC patients have aberrant Wnt signaling [5]. Wnt target genes such as SNAI1 [6, 7], ZEB1 [8] and Twist [9] are master inducers of the epithelial-mesenchymal transition (EMT) program. As the first step of cancer metastasis, EMT promotes the initiation of CRC metastasis [10]. Additionally, the Wnt signaling pathway can regulate adult intestinal stem cells (ISCs) homeostasis, and aberrant activation induces the development of CRC stem cells [11]. The properties of cancer stem cells, including plasticity and better adaptation to the microenvironment, promote CRC metastasis [12].

The rhomboid family is a protein family conserved through many taxonomic kingdoms in nature [13]. This protein family physiologically functions as an intramembrane serine protease [14] that mainly consists of active proteases and inactive members lacking catalytic residues [15]. Rhomboid family proteins have been proven to regulate growth factor signaling, mitochondrial dynamics, inflammation, parasite invasion, and the machinery of protein quality control [14]. Although the rhomboid family is a relatively well studied group of proteins, elucidating the function of the active mammalian analogs is ambitious. Rhomboid domain containing 1 (RHBDD1) is a mammalian member of this protein family and the 6th intramembrane serine protease [14]. As one of the mammalian members, RHBDD1 has a much clearer picture of its function. Our group showed that RHBDD1 can promote CRC growth through the EGFR pathway [16]. RHBDD1 can cleave the Bcl2 family member BIK [17] and polytopic membrane protein TSAP6 [18]. Other researchers showed that RHBDD1 participates in ER-associated degradation (ERAD) [19] and triggers ER export and non-canonical secretion of membrane-anchored TGFα [20]. Researches on RHBDD1 facilitate our understanding about rhomboid family proteins.

Few studies have proven that rhomboid family proteins can regulate cancer metastasis. RHBDD2 is demonstrated overexpressed in the advanced stages of breast and colorectal carcinomas [21, 22], but the relative mechanism is completely unknown. Here, we found that RHBDD1 can promote CRC metastasis. The present study showed that RHBDD1 can increase the levels of p552-β-catenin and p675-β-catenin to up-regulate Wnt signaling pathway activity. The Wnt target gene ZEB1 is increased by RHBDD1, which induces CRC to undergo the EMT program, causing the metastatic phenotype in vivo and in vitro. Our results provide a new mechanism of rhomboid family proteins to promote tumor metastasis. As a membrane protein, RHBDD1 has the potential and advantage to become a new therapeutic target or clinical biomarker for metastatic CRC.

## Methods

### Cell culture and reagents

HCT-116, HCT-8, CACO-2, and DLD1 cells were all obtained from and authenticated by the Cell Resource Center at Peking Union Medical College. All these cell lines were passaged fewer than 6 months after purchase for all the experiments. HCT-116, and DLD1 cells were cultured in IMDM (HyClone, SH30228.01) supplemented with 10% fetal bovine serum (Gibco, 10099141); HCT-8 cells were cultured in RPMI-1640 medium (Cell Resource Center, IBMS, CAMS/PUMC, CCCM019) supplemented with 10% fetal bovine serum; and CACO-2 cells were cultured in MEM-EBSS medium (HyClone, SH30024.01) supplemented with 10% fetal bovine serum and nonessential amino acids (HyClone, SH30238.01). Expression plasmids for RHBDD1, S552D-β-catenin and S675D-β-catenin were cloned into a pcDNA6.0 plasmid with a C-terminal myc-tag. S552D-β-catenin and S675D-β-catenin point mutation constructs were generated using the overlap extension PCR method. The primers used are shown in the Additional file 1: Table S1. The sequences of the two RNAi oligonucleotides targeting RHBDD1 are shown in the Additional file 1: Table S1 and were generated by GenePharma. CHIR99021 was purchased from Selleck Chemicals (S2924) and was dissolved in DMSO. Puromycin was purchased from MP Biomedicals (194539). The transfection regent Lipo3000 was purchased from Invitrogen (L3000008).

### Tissue analyses

A total of 8 histopathologically confirmed CRC tissues at the Cancer Hospital, Chinese Academy of Medical Sciences were used for western blot analysis. Prior patient consent and approval from the Institutional Research Ethics Committee were obtained for the use of these patient specimens for research purposes. The study was conformed to the ethical guidelines of the Declaration of Helsinki. Gray scale scanning was conducted using ImageJ software. The tissue microarray comprising tumor tissues and their corresponding adjacent normal tissues from 71 patients with CRC were obtained from Shanghai Biochip (HCol-Ade150CS-01). The protein expression levels were evaluated using an immunostaining score, which was calculated as the sum of the proportion and intensity of the stain. Briefly, a proportion score was assigned first, which represented the estimated percentage of positively stained tumor cells (0, < 1%; 1, 1–24%; 2, 25–49%; 3, 50–74%; and 4, 75–100%). Next, an intensity score was assigned, which represented the average intensity of the positively stained tumor cells (0, none; 1, weak, 2, intermediate; and 3, strong). The proportion and intensity scores were then added to obtain a final score, which ranged from 0 to 7.

### Antibodies

The anti-RHBDD1 mouse monoclonal antibody was prepared in-house. Antibodies against β-catenin (8480), phospho-33/37/41-β-catenin (9561), phospho-552-β-catenin (5651), phospho-675-β-catenin (4176), ZEB1 (3396), and β-actin (4970) were purchased from Cell Signaling Technology. Antibodies against Lamin A/C (2966-1) were purchased from Epitomics. Antibodies against the myc-tag (M5546) and tubulin (T6199) were purchased from Sigma-Aldrich. Antibodies against the GAPDH (TA-08) was purchased from ZSGB-BIO.

### RHBDD1 mutant and rescued HCT-116 stable cell lines

The RHBDD1 mutant stable cell line was established in our previous work [16]. The RHBDD1 rescue HCT-116 stable cell line was derived from the HCT-116 mutant stable cell line. The mutant cell line was infected with either lentivirus-RHBDD1 or lentivirus-control. Double dilutions of the cells were seeded in 96-well cell culture plates to generate single cell-derived clones. The positive clones were selected by immunoblotting for RHBDD1. The selected clones were expanded, cultured and preserved for subsequent experiments.

### Targeted crispr-cas9 RHBDD1-KO mutant HCT-116 cell line

An sgRNA (TAGCAACTTTGGCCCTCAAC) was designed using the Zhang lab website (http://www.genome-engineering.org). Off-target effects were predicted using the Cas-OFFinder website (http://www.rgenome.net/cas-offinder). The primers used were as follows: Forward: ATGCAACGGAGATCAAGAGG, Reverse: TGTCTCCCTTACCTGAGAAACC. The experiment was conducted according to methods described by Cong et al. [23]. The plasmid used was purchased from Addgene (42230).

### RHBDD1 knockdown HCT-116 stable cell line

The wild-type HCT-116 cell line was transduced with lentivirus-luciferase to generate an HCT-116-luciferase stable cell line. Then, this cell line was used to establish an HCT-116 RHBDD1 knockdown stable cell line. The HCT-116-luciferase stable cell line was transduced with lentivirus-Negative control, lentivirus-Si-RHBDD1-1#, or lentivirus-Si-RHBDD1-2#. Double dilutions of the cells were seeded in 96-well cell culture plates to generate single cell-derived clones. The positive clones were selected based on GFP expression followed by expansion, culturing and preservation for subsequent experiments.

### Cell migration and invasion assay

For the cell migration assay, 1 × 10^5^ cells in serum-free IMDM were seeded in the upper chamber of the transwell insert (Millipore, MCEP24H48), and the lower chamber contained complete IMDM with 10% FBS. At 24 h after inoculation, the transwell chamber was collected and stained with crystal violet. For the cell invasion assay, the upper chamber of the transwell insert was pre-coated with Matrigel (BD, 356234), and incubated at 37 °C for 3 h to form the Matrigel layer in the chamber. A total of 2 × 10^5^ cells in serum-free IMDM were seeded in the upper chamber of the transwell insert, and the lower chamber contained complete IMDM with 20% FBS. At 24 h after inoculation, the transwell chamber was collected and stained with crystal violet. The experiment was performed in triplicate.

### In vivo tumor metastasis

Animal experiments were performed with the approval of the Peking Union Medical College Animal Care and Use Committees. Male and female NOD/SCID mice (6 weeks old) were used to perform the metastasis analysis in vivo. Three stable cell lines (HCT-116-nc-luciferase, HCT-116-Si-RHBDD1-1#-luciferase and HCT-116-Si-RHBDD1-2#-luciferase; *n* = 4 per cell type) were inoculated via tail vein injection (5 × 10^5^ cells per mouse). At 28 days after injection, the luciferase activity and the signal distribution in each mouse was detected using an in vivo imaging system.

### RNA sequencing

The RNA sequencing was performed in triplicate in each group. Total RNA was isolated from HCT-116 cells transfected with either Si-RHBDD1-pool or Control Si-RNA. The isolated RNAs were subjected to quality control, mRNA library establishment and Illumina HiSeq sequencing. HTSeq software was used to get the gene expression levels. And then FPKM of each gene was calculated based on the length of the gene and reads count mapped to this gene. Genes with a padj< 0.05 found by DESeq were assigned as differentially expressed. The KEGG pathway enrichment analysis result is shown as the adjusted *P* value (padj), differential gene count and gene ratio (differential gene count in this pathway versus total differential gene count). A heatmap analysis is shown as normalized gene expression (FPKM).

### Dual-luciferase reporter assay

The TCF/LEF binding regions were used for the canonical Wnt signaling pathway. HCT-116 cells were seeded in a 24-well cell culture plate and co-transfected with the pGL3-Basic plasmid containing the specific promoter (200 ng/well) and the pRL-TK plasmid (10 ng/well). At 36–48 h later, the cells were analyzed for fluorescence intensity using a Dual-Luciferase Reporter Assay System (Promega, E1910). The cells were washed twice with pre-chilled PBS, lysed with 100 μl of PLB per well for 15 min at room temperature, and transferred to a 96-well plate (Corning, 3917) (15 μl lysate/well) for luminescence detection. The results are shown as the ratio of firefly luciferase intensity and renilla luciferase intensity. The experiment was performed in triplicate.

### TOP/FOP flash reporter assay

HCT-116 cells were seeded in a 24-well cell culture plate and co-transfected with the pRL-TK plasmid (10 ng/well) and either TOP flash plasmid or FOP flash plasmid (200 ng/well). At 36–48 h later, the cells were analyzed using Dual-Luciferase Reporter Assay System (Promega, E1910) to assess the luminescence intensity. The exact procedures performed in this experiment were the same as those for the dual-luciferase reporter assay. The results are shown as the ratio of TOP Flash activity and FOP Flash activity. The experiment was performed in triplicate.

### Real-time PCR

Total RNA was isolated from the different cell lines using TRIzol Reagent (Invitrogen, 15596018) according to the manufacturer’s instructions. Equal amounts of RNA were reverse transcribed into cDNA using a Transcriptor First Strand cDNA Synthesis Kit (Roche, 04896866001) as instructed by the manufacturer. Quantitative PCR was performed using a ABI Step-One Plus system. PCR reactions were carried out in 10-μl reactions using TransStart Top Green qPCR SuperMix (TransGen Biotech, AQ131–02) and 0.5 mM specific primers. The primers used for PCR are shown in the Additional file 1: Table S1.

### Isolation of cytosolic/nuclear proteins

Cytosolic and nuclear protein extraction was performed using a Nuclear/Cytosol Fractionation Kit (BioVision, K266-25) according to the manufacturer’s instructions. Each fraction was tested for the presence of the cytosolic marker GAPDH and the nuclear marker Lamin A/C by western blotting as appropriate.

### Statistical analyses

The data are reported as the mean and SEM. All statistical analyses were performed using Prism version 5.0 for Windows. The data were analyzed for the normality of distribution using the Kolmogorov–Smirnov normality test (*n* > 50) or the Shapiro–Wilk normality test (*n* ≤ 50). *P* > 0.05 indicated that the data were normal. The Pearson correlation was performed on the normality distribution and the variance similar data, and the Spearman correlation was performed on the stratified or the variance heterogeneity data. Fisher’s exact test was used for analyzing the proportions between two groups. An unpaired t test was used to compare between two groups. One-way ANOVA was performed for comparisons among more than two groups. *P* < 0.05 (two-tailed) was considered statistically significant. *P* < 0.05 was marked as ‘*’, *P* < 0.01 was marked as ‘**’ and *P* < 0.001 was marked as ‘***’.

## Results

### RHBDD1 expression correlates with CRC metastasis

**Fig. 1:**
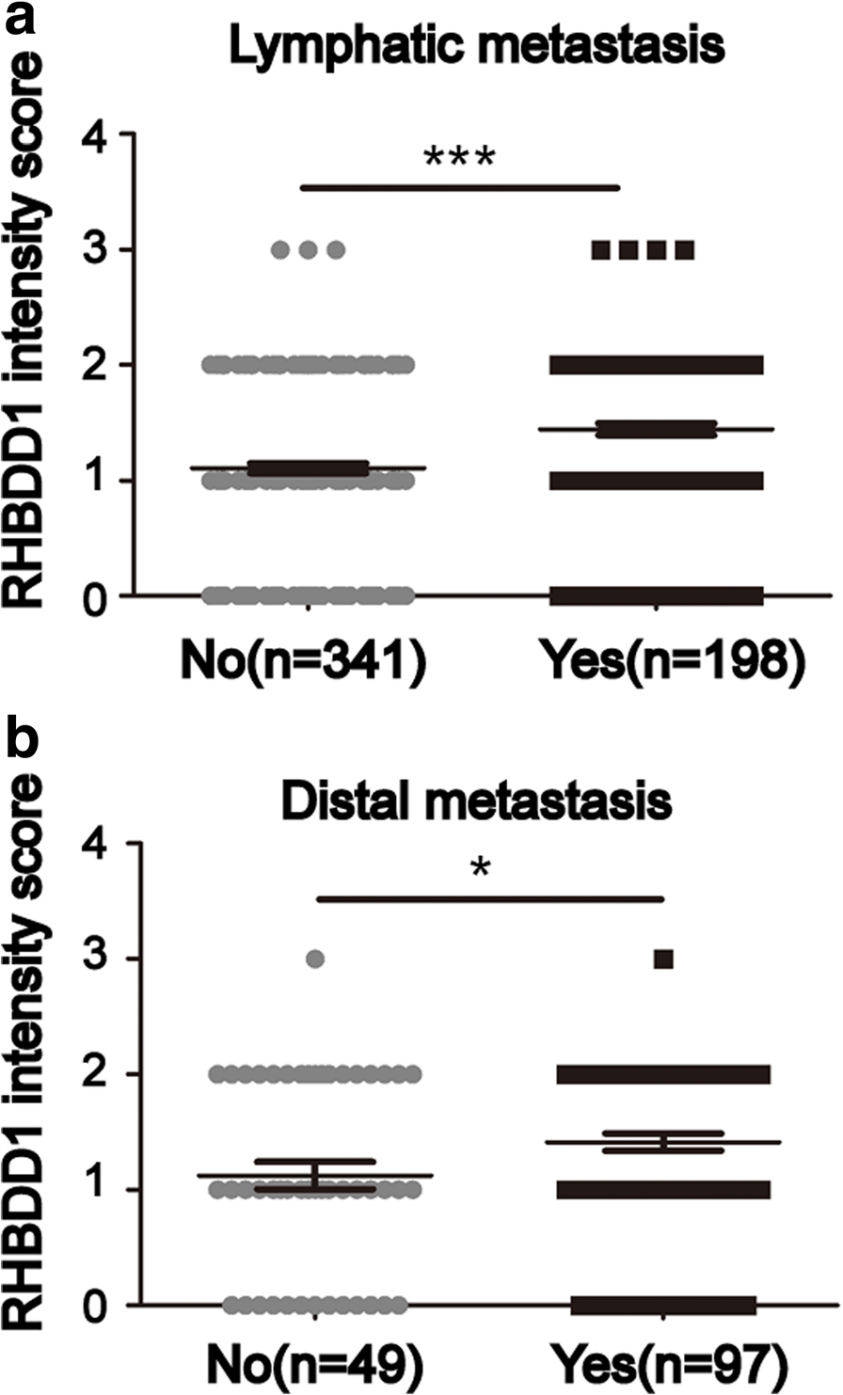
RHBDD1 expression correlates with CRC lymphatic metastasis and distal metastasis. **a**. Analysis of 539 patients of RHBDD1 intensity scores in tissues with or without lymphatic metastasis. No, N0; Yes, N1 and N2. ***, *P* < 0.001. **b**. Analysis of 146 patients of RHBDD1 intensity scores in tissues with or without distal metastasis. No, local recurrence and implantation metastasis; Yes, distal metastasis. *, *P* < 0.05. **a**. **b**, Data are mean ± SEM

A previous study by our group performed a tissue microarray analysis of 539 colorectal tumor tissues [16]. We reanalyzed the correlation between RHBDD1 and metastatic parameters in the same cohort recently. We assigned intensity score 0 or 1 as negative, and 2 or 3 as positive. In 539 patients, the percentage of patients with positive RHBDD1 expression is higher (*P* < 0.001) among those with lymphatic metastasis than patients without lymphatic metastasis (Table 1), and the intensity score of RHBDD1 is also significantly higher (*P* < 0.001) in patients with lymphatic metastasis (Fig. 1a). A total of 146 patients in the cohort have records regarding the presence of local recurrence, implantation metastasis or distal metastasis. Among this patient subset, the percentage of individuals positive for RHBDD1 expression is higher (*P* < 0.05, one-tailed, other *P* values in the article are all two-tailed) in patients with distal metastasis than in patients with local recurrence and implantation metastasis (Table 1), and the intensity score of RHBDD1 is also significantly higher (*P* < 0.05) in patients with distal metastasis (Fig. 1b). Therefore, RHBDD1 expression positively correlates with CRC metastatic parameters.

**Table 1:** Association between RHBDD1 expression and metastatic parameters in colorectal cancer patients

Metastatic parameters	N	RHBDD1 expression		*P* value^a^
		negative	positive	
Lymphatic metastasis				*P* < 0.001
No	341	220(65%)	121(35%)	
Yes	198	92(46%)	106(54%)	
Distal metastasis				*P* < 0.05^b^
No	49	31(63%)	18(37%)	
Yes	97	44(45%)	53(55%)	

Lymphatic metastasis, No, N0; Yes, N1 and N2; Distal metastasis, No, local recurrence and implantation metastasis; Yes, distal metastasis

^a^Fisher’s exact test was used for obtaining the *P* value

^b^One-tailed (other *P* values in the article are all two-tailed)

### RHBDD1 expression increases colorectal cancer cells metastasis in vivo and in vitro

**Fig. 2:**
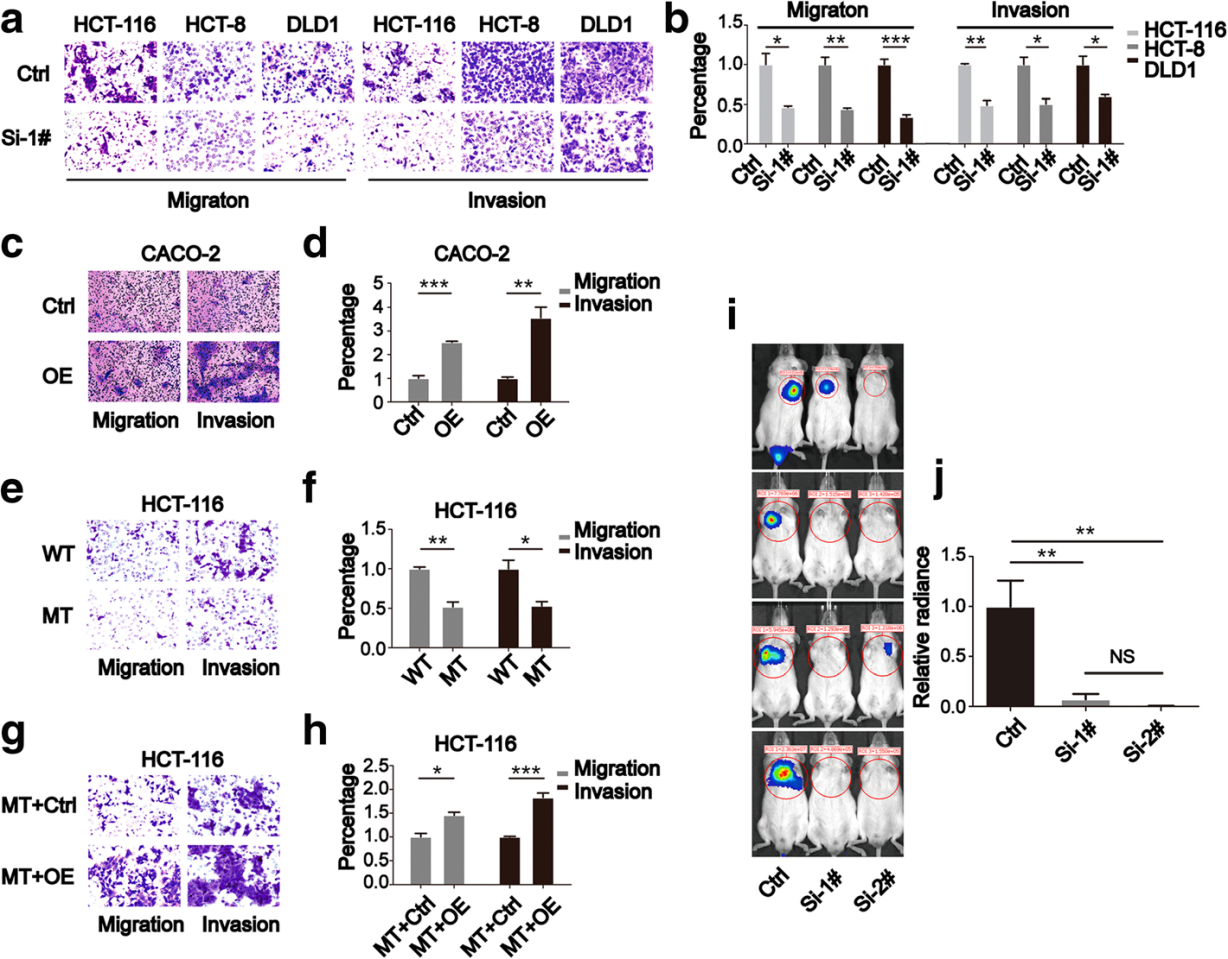
RHBDD1 could affect the metastatic ability of colorectal cancer cells in vivo and in vitro. **a, b**. Images and statistics of the migration assay and invasion assay when RHBDD1 is knocked down in HCT-116, HCT-8 and DLD1 cells. For migration assay, 1 × 10^5^ cells were seeded in the upper chamber of the transwell insert, as described in Methods section. For invasion assay, the upper chamber of the transwell insert was pre-coated with Matrigel to form the Matrigel layer in the chamber. A total of 2 × 10^5^ cells were seeded in the upper chamber of the transwell insert as described in Methods section.× 100 magnification, cells were counted and calculated. Ctrl, control; Si-1#, Si-RHBDD1–1#; Si-2#, Si-RHBDD1–2#. **c, d**. Images and statistics of the migration and invasion assays when RHBDD1 is over-expressed in CACO-2 cells. RHBDD1 was cloned into a pcDNA6.0 plasmid with a C-terminal myc-tag. 2 μg plasmids were transfected for each well of 6-well cell culture plate. OE, over-expression. **e, f**. Images and statistics of the migration and invasion assays in HCT-116 cells expressing wild-type and mutant RHBDD1. WT, wild-type; MT, mutant. **g, h**. Images and statistics of the migration and invasion assays in HCT-116 cells when wild-type RHBDD1 expression is rescued. MT + Ctrl, mutant cell line infected with lentivirus-control; MT + OE, mutant cell line infected with lentivirus-RHBDD1. **a-h**. The experiments were performed in triplicate. **i**. In vivo imaging of metastasis in NOD/SCID mice injected with HCT-116 cells with stable knockdown of RHBDD1 or control Si-RNA. NOD/SCID mice were inoculated via tail vein injection (5 × 10^5^ cells per mouse). Twenty-eight days later, the luciferase activity and the signal distribution in each mouse was detected. **j**. Statistical analysis of luminescence in **i**. The data in each column were calculated from 4 mice. All data are mean ± SEM, *, *P* < 0.05; **, *P* < 0.01; ***, *P* < 0.001, NS, not significant

We analyzed the protein expression profile of RHBDD1 in 4 CRC cell lines. HCT-116, DLD1, and HCT-8 cells have higher levels of RHBDD1 protein expression (Additional file 2: Figure S1A), and CACO-2 cells have lower levels of RHBDD1 protein expression (Additional file 2: Figure S1A); thus, these cell lines were employed for further study. HCT-116, HCT-8, and DLD1 cells showed decreased migration (*P* < 0.05, *P* < 0.01, *P* < 0.001) (Fig. 2a, b) and invasion (*P* < 0.01, *P* < 0.05, *P* < 0.05) (Fig. 2a, b) upon RHBDD1 knockdown. CACO-2 cells showed increased migration (*P* < 0.001) and invasion (*P* < 0.01) when RHBDD1 was ectopically over-expressed (Fig. 2c, d). We used an RHBDD1 mutant stable cell line, which degraded endogenous RHBDD1 via the proteasome [16], to analyze its metastatic ability. RHBDD1-mutant HCT-116 cells showed decreased migration (*P* < 0.01) and invasion (*P* < 0.05) abilities (Fig. 2e, f). The RHBDD1 mutant cell line was separately infected with either RHBDD1 lentivirus or control lentivirus to obtain the RHBDD1 rescued stable cell line. This stable rescue clone showed increased migration (*P* < 0.05) and invasion (*P* < 0.001) abilities compared to the control clone (Fig. 2g, h). These data indicate that RHBDD1 can promote CRC cell metastasis in vitro. Then, we employed an in vivo imaging system to analyze whether RHBDD1 can promote CRC metastasis in vivo. After injection of HCT-116 cells with stable RHBDD1 knockdown via the caudal vein, we observed that pleural metastasis was significantly less severe (*P* < 0.01) compared to that observed upon injection with control cells (Fig. 2i, j).

### Depression of RHBDD1 attenuates Wnt signaling pathway activity and influences β-catenin protein expression

**Fig. 3:**
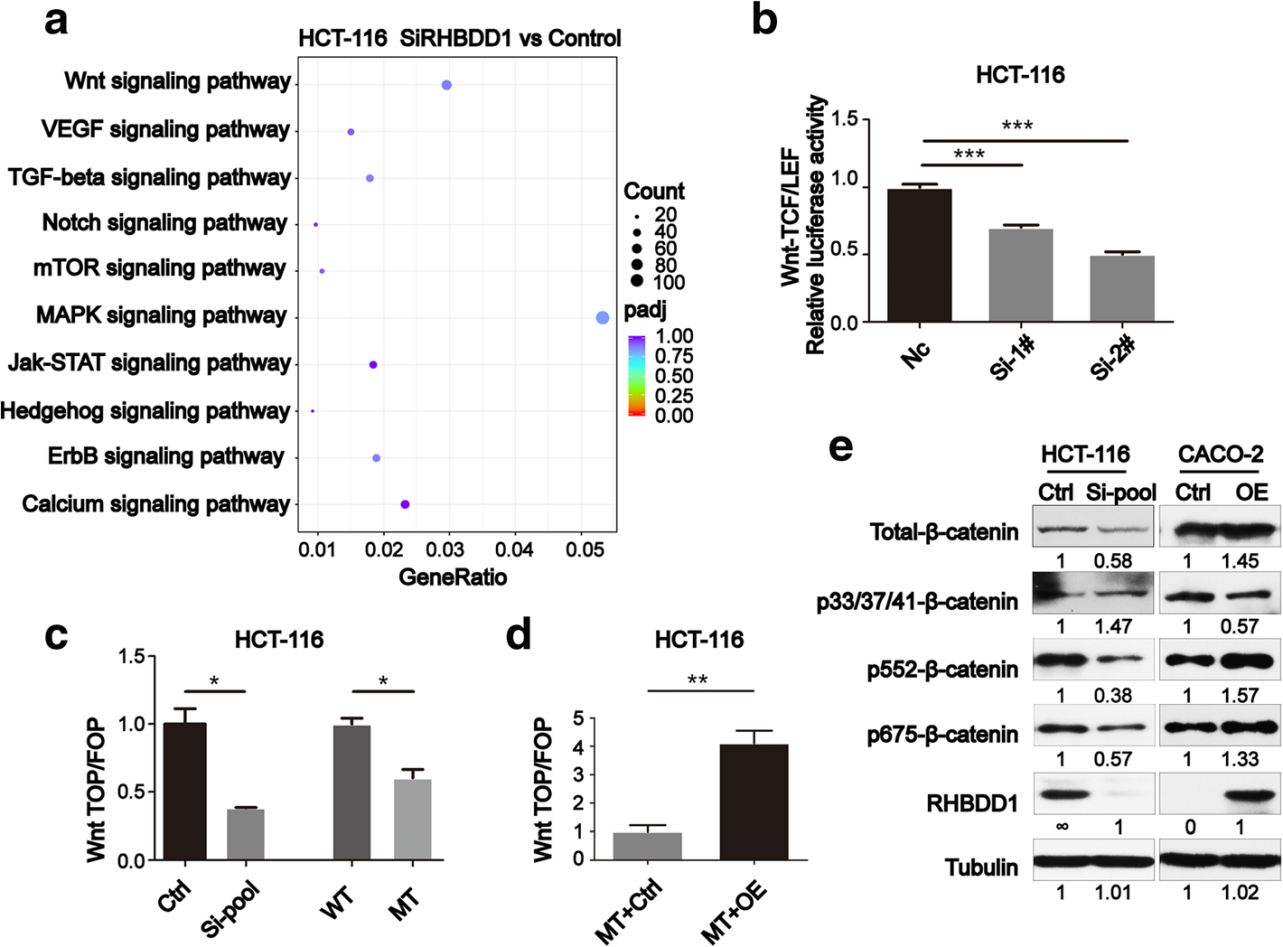
Depression of RHBDD1 attenuates Wnt signaling pathway activity and influences β-catenin protein expression. **a**. Top 10 signaling pathway based on KEGG enrichment analysis of signal transduction pathway sub-categories. Count, differential gene count in indicated pathway; padj, adjusted *P* value of indicated pathway; GeneRatio, differential gene count in indicated pathway versus total differential gene count. RHBDD1 was knocked down with Si-RHBDD1-pool, mixture of Si-RHBDD1–1# and Si-RHBDD1–2# in a ratio of 1:1. **b**. Dual-luciferase reporter assay of Wnt signaling pathway. The TCF/LEF binding regions were used as indicated in the Methods section. Ctrl, control; Si-1#, Si-RHBDD1–1#; Si-2#, Si-RHBDD1–2#; ***, *P* < 0.001. **c, d**. TOP/FOP flash reporter assay in cells with RHBDD1 knockdown (**c**), mutant RHBDD1 (**c**) and rescued wild-type RHBDD1 (**d**), respectively. The TOP flash plasmid or FOP flash plasmid and pRL-TK plasmid were used as indicated in the Methods section. Ctrl, control; Si-pool, mixture of Si-RHBDD1–1# and Si-RHBDD1–2# in a ratio of 1:1; WT, wild-type; MT, mutant; MT + Ctrl, mutant cell line infected with lentivirus-control; MT + OE, mutant cell line infected with lentivirus-RHBDD1. The experiments in **b**, **c**, and **d** were performed in triplicate. *, *P* < 0.05; **, *P* < 0.01; ***, *P* < 0.001. **e**. Immunoblot analysis of RHBDD1, total β-catenin and phosphorylated β-catenin in cells with knocked down and over-expressed RHBDD1. Tubulin was used as a loading control. Bands were quantified with ImageJ software

We conducted RNA-Seq analyses to find the possible signaling pathway by which RHBDD1 promotes CRC metastasis. RNA from either control HCT-116 cells or HCT-116 cells with RHBDD1 knockdown were isolated and analyzed. Differential expression genes in the two groups were analyzed and enrichened in KEGG pathway database. To elucidate the regulatory mechanism of the signaling transduction of metastasis, we focused on the signal transduction pathway sub-categories from the KEGG pathway database. The pathways in this category were ranked, and the top 10 are shown in Fig. 3a. As shown in the figure, the MAPK signaling pathway had the largest differential gene counts, which was already proven to be influenced by RHBDD1 to regulate CRC growth [16]. These data showed the reliability of the RNA-Seq results. The Wnt signaling pathway had the second largest differential gene counts, which showed the possible role of this pathway in regulating CRC metastasis. We used a dual-luciferase reporter assay to further verify the KEGG enrichment results. The TCF/LEF binding regions were used. The results showed that the canonical Wnt signaling pathway exhibited significant decreases (*P* < 0.001) in activity when RHBDD1 is knocked down (Fig. 3b). To further validate the Wnt signaling pathway, we conducted the TOP/FOP flash reporter assay. The TOP flash plasmid has 7 TCF/LEF binding sites and the FOP flash plasmid has 6 mutated TCF/LEF binding sites. The ratio of TOP and FOP luciferase activity is analyzed. The results showed that RHBDD1 knockdown can decrease (*P* < 0.05) pathway activity (Fig. 3c), stable mutant RHBDD1 can decrease (*P* < 0.05) pathway activity (Fig. 3c), whereas rescuing wild-type RHBDD1 expression can restore (*P* < 0.01) pathway activity (Fig. 3d).

The core protein in the Wnt signaling pathway is β-catenin. In addition, the primary method of regulating β-catenin is phosphorylation. Because p33/37/41/45-β-catenin, p552-β-catenin and p675-β-catenin are directly associated with Wnt-signaling pathway activity, we analyzed the total and phosphorylated protein levels of β-catenin. The results (Fig. 3e) showed that the total-, p552- and p675-β-catenin, which were proved to activate β-catenin-mediated TCF signaling, decreased in HCT-116 cells with knockdown of RHBDD1 than the control group; while p33/37/41-β-catenin, which led to β-catenin degradation by the proteasome, increased in HCT-116 cells with knockdown of RHBDD1 than the control group. In CACO-2 cells, the expression pattern of all β-catenin was reversed upon over-expressed RHBDD1. The β-catenin expression pattern influenced by RHBDD1 is coincident with the TOP/FOP analysis. Thus, we confirmed that RHBDD1 can regulate Wnt signaling pathway activity and the protein levels of β-catenin.

### RHBDD1 influences Wnt signaling pathway activity by regulating the phosphorylation of ser552 and ser675 of β-catenin

**Fig. 4:**
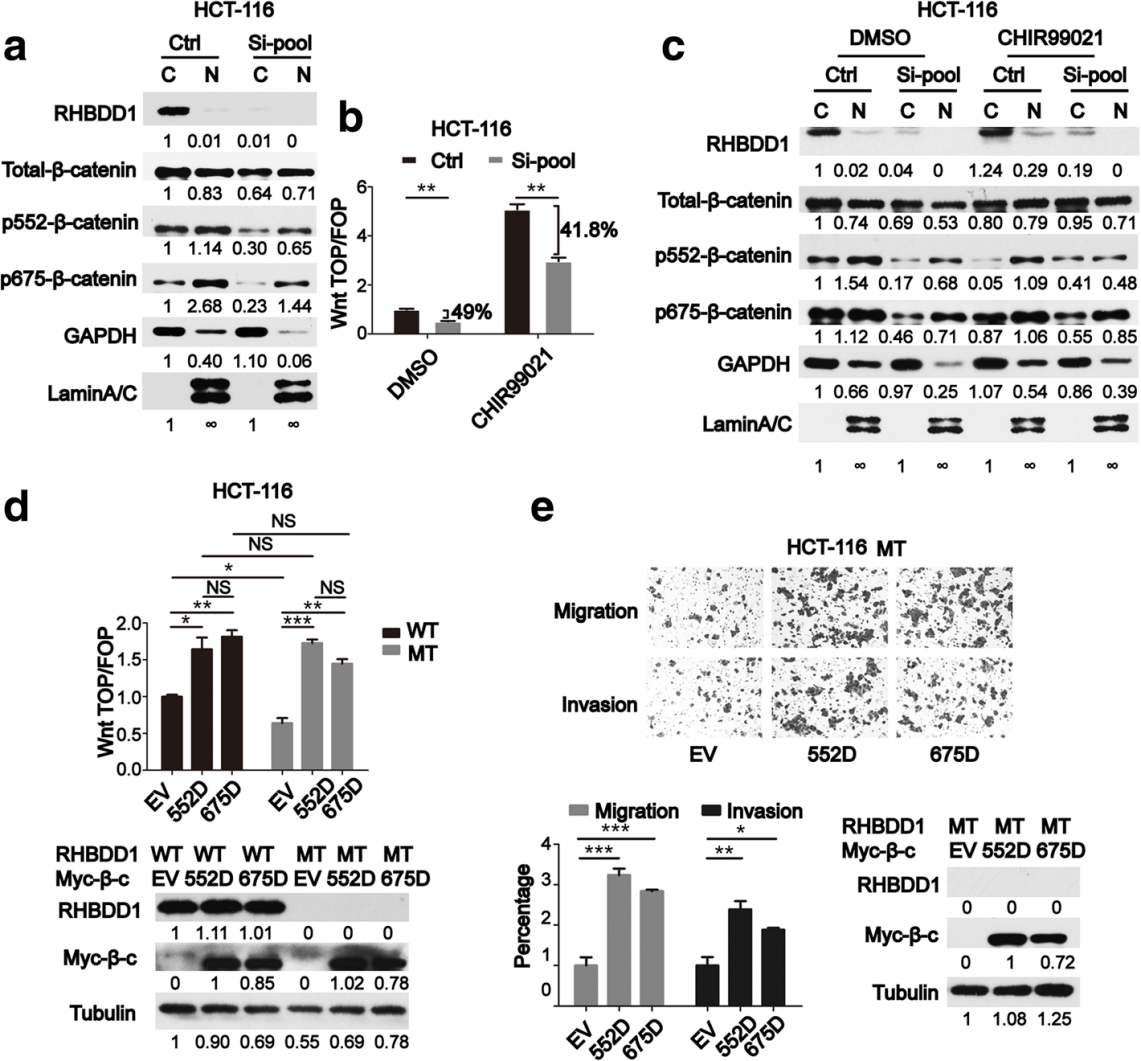
RHBDD1 influences Wnt signaling activity by regulating the phosphorylation of ser552 and ser675 of β-catenin. **a**. Immunoblot analysis of RHBDD1, total β-catenin and indicated phosphorylated β-catenin in the isolated cytosolic and nuclear lysates from HCT-116 cells transfected with RHBDD1 Si-RNA and control Si-RNA. GAPDH was used as the cytosol marker, and Lamin A/C was used as the nuclear marker. Ctrl, control; Si-pool, mixture of Si-RHBDD1–1# and Si-RHBDD1–2# in a ratio of 1:1; C, cytosol; N, nucleus. Bands were quantified with ImageJ software. **b**. TOP/FOP flash reporter assay of HCT-116 cells stimulated with CHIR99021. DMSO was used as the solvent control. HCT-116 cells were transfected with Control and RHBDD1 Si-RNA, and 48 h later, cells were stimulated with 100 ng/ml CHIR99021 or solvent control DMSO dissolved in serum free culture medium for 24 h, then cells were harvested for luciferase analysis. **c**. Immunoblot analysis of isolated cytosolic and nuclear protein fractions from HCT-116 cells stimulated with CHIR99021. **d**. Wild-type HCT-116 cells and HCT-116 cells stably expressing mutant RHBDD1 were transfected with empty vector, S552D-β-catenin or S675D-β-catenin, and the TOP/FOP flash reporter assay was performed. Immunoblot analysis of RHBDD1 and β-catenin in transfected HCT-116 cells. Tubulin was used as a loading control. WT, wild-type; MT, mutant; EV, empty vector; 552D, S552D mutant construct; 675D, S675D mutant construct. **e**. HCT-116 cells stably expressing mutant RHBDD1 were transfected with empty vector, S552D-β-catenin or S675D-β-catenin and subjected to cell migration and invasion assays. Images of representative wells of cell migration and invasion assay were shown, cells were counted and calculated. Immunoblot analysis of RHBDD1 and β-catenin in transfected HCT-116 cells was performed. **a, c, d, e**. Bands were quantified with ImageJ software. The experiments in **b, d**, and **e** were performed in triplicate. All data are mean ± SEM.*, *P* < 0.05; **, *P* < 0.01; ***, *P* < 0.001; NS, not significant

A critical step of Wnt signaling pathway activation is the translocation of β-catenin from the cytosol to the nucleus. In the nucleus, it can form a complex with TCF/LEF to initiate transcription of downstream genes [11]. We performed cytosolic/nuclear protein isolation and analyzed the distribution of total and phosphorylated β-catenin between the cytosol and the nucleus. In HCT-116 cells with knockdown of RHBDD1, the total and phosphorylated β-catenin expression levels were all decreased in either the cytosol or the nucleus, while the percentages of the cytosol and the nucleus remained relatively stable (Fig. 4a). It seemed that RHBDD1 can influence the overall protein expression of total-, p552- and p675-β-catenin but cannot influence the translocation process. CHIR99021 can potently inhibit GSK3α/β and stimulation with this molecule can protect β-catenin from ubiquitin-associated degradation. We stimulated HCT-116 cells with CHIR99021 after knocking down RHBDD1, and the TOP/FOP flash reporter assay results showed that Wnt signaling pathway activity was still decreased significantly (*P* < 0.01) compared with stimulated control (Fig. 4b), with a slightly recovery of 7.2% compared with the DMSO treated group. We stimulated HCT-116 cells with CHIR99021 after knocking down RHBDD1, and conducted western blot analyses of total and phosphorylated β-catenin expression after cytosolic and nuclear isolation (Fig. 4c). The results showed that CHIR99021 can rescue β-catenin expression in either the cytosol or the nucleus than the control group. Fig. 4b and Fig. 4c indicate that total-β-catenin is not the key effector of RHBDD1-mediated regulation of the Wnt signaling pathway activity. At the meantime, the levels of ser552 and ser675 phosphor-β-catenin kept decreasing (Fig. 4c) after knocking down RHBDD1 compared with the control group, which may result in the decrease of pathway activity.

We established β-catenin constructs with point mutations of serine 552 and 675 to the phosphomimetic aspartic acid [24], positive mutation of phosphor-β-catenin at residues 552 and 675. HCT-116 cells with either mutant or wild-type RHBDD1 were transfected with control, S552D, or S675D plasmids (Fig. 4d). The TOP/FOP flash reporter assay showed that upon transfection with control plasmid, the pathway activity decreased significantly (*P* < 0.05) in HCT-116 cells with mutant RHBDD1 compared with in wild-type HCT-116 cells, which was the repetition of Fig. 3c. However, upon transfection with either S552D or S675D constructs, the pathway activity increased significantly in either HCT-116 cells with mutant RHBDD1 (*P* < 0.001, *P* < 0.01) or in wild-type HCT-116 cells (*P* < 0.05, *P* < 0.01). The results (Fig. 4d) showed that in HCT-116 cells with mutant RHBDD1, upon transfection with either S552D or S675D constructs, the pathway activity can be recovered to levels comparable to those in wild-type HCT-116. Additionally, In HCT-116 cells with mutant RHBDD1, the migration and invasion assays (Fig. 4e) showed that the metastatic ability can be recovered significantly by over-expressing the S552D (*P* < 0.001, *P* < 0.01) and S675D (*P* < 0.001, *P* < 0.05) constructs. Collectively, these data proved that the pathway activity is directly associated with p552- and p675-β-catenin and that RHBDD1 regulates the pathway activity mainly through these two phosphorylated residues on β-catenin.

### RHBDD1 influences the EMT and stemness signatures of HCT-116 cell line

**Fig. 5:**
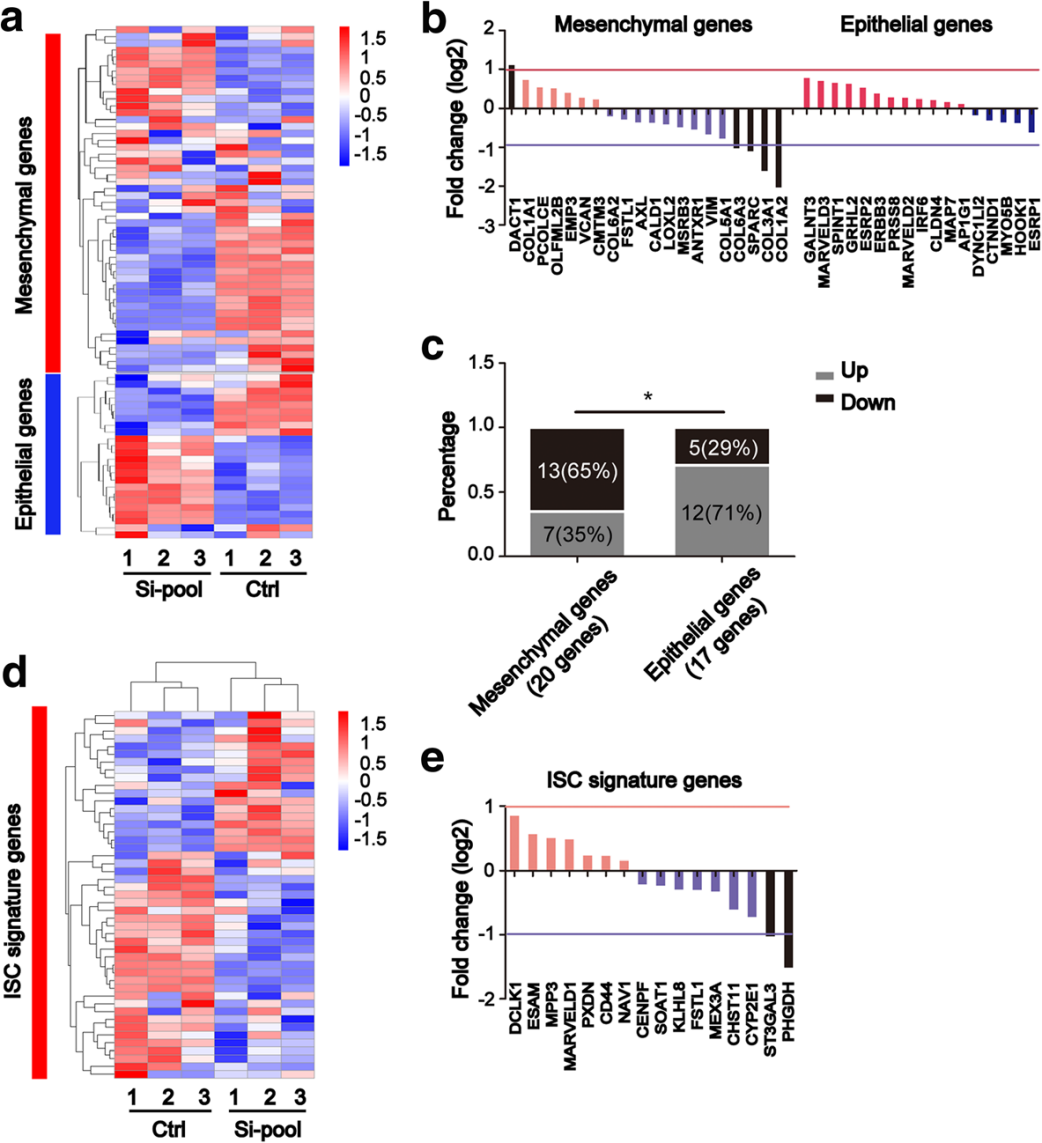
RHBDD1 influences the EMT and stemness signatures of HCT-116 cell line. **a**. Heatmap analysis of the pan-EMT signature in RHBDD1 knockdown and control HCT-116 cells. Mesenchymal genes are shown in the upper half, and the epithelial genes are shown in the bottom half. 1, 2, 3, different replicates. Ctrl, control; Si-pool, mixture of Si-RHBDD1–1# and Si-RHBDD1–2# in a ratio of 1:1. **b**. Differential expression genes in pan-EMT signature were analyzed. The Red line above the X-axis represents log2FoldChange > 1; the blue line beneath the X-axis represents log2FoldChange < − 1. Genes with log2FoldChange > 1 or log2FoldChange < − 1 were shown in black. **c**. Fisher's exact test of differential expression genes in mesenchymal genes and epithelial genes. Numbers (percentages) in the rectangles represent the numbers (percentages) of differential expression genes in indicated groups. *, *P* < 0.05. **d**. Heatmap analysis of the ISC signature in RHBDD1 knockdown and control HCT-116 cells. **e**. Differential expression genes in ISC signature were analyzed

The Wnt signaling pathway regulates EMT and ISCs homeostasis. Lauren Averett Byers’ group reported a pan-cancer EMT signature derived from 11 cancer types in 2016 [25]. They used four established EMT markers—CDH1 (epithelial marker, E type), CDH2 (mesenchymal marker, M type), VIM (M type), and FN1 (M type)—as seeds to develop a pan-cancer EMT signature on the basis of TCGA pan-cancer RNA-Seq data. The derived 77 signature genes were identified in our RNA-Seq results, and a heatmap analysis was constructed (Fig. 5a). Seventy-four genes in the signature with calculated FPKM as indicated in the Methods section were shown in the heatmap. The RNA sequencing was performed in triplicate in HCT-116 cells transfected with RHBDD1 Si-RNA or control scramble Si-RNA. We assigned the adjusted *P* value (padj) < 0.05 found by DESeq as differentially expressed. Then differentially expression genes in the EMT signature were identified and analyzed (Fig. 5b). In mesenchymal genes, 13 genes decreased significantly upon knocking down RHBDD1, with 4 genes have log2FoldChange < − 1; 7 genes increased significantly, with 1 gene have log2FoldChange > 1. In epithelial genes, 12 genes increased significantly upon knocking down RHBDD1, and 5 genes decreased significantly. The proportions of up and down regulated differential expression genes in mesenchymal genes and in epithelial genes were significantly different (*P* < 0.05) (Fig. 5c). The results showed that knockdown of RHBDD1 led to a decreasing trend in the mesenchymal gene expression pattern. In addition, the epithelial gene expression pattern tended to increase. This result indicated that RHBDD1 was positively correlated with EMT and could maintain the mesenchymal phenotype in colorectal cell lines; this mesenchymal phenotype endowed the cells with metastatic potential. Eduard Batlle reported an ISC signature in 2011 [26]. They isolated ISCs using EphB2, which is expressed at high levels in the ISC membrane. Then, they derived an ISC signature based on EphB2 expression level, thus creating a list of 71 probes (54 annotated genes). We identified the 54 genes in our RNA-Seq results and constructed a heatmap analysis (Fig. 5d). Forty-seven genes in the signature with calculated FPKM were shown in the heatmap. Differential expression genes in the ISC signature were also identified and analyzed (Fig. 5e). Nine genes decreased significantly upon knocking down RHBDD1, with 2 genes have log2FoldChange < − 1; 7 genes increased significantly, but none of them met log2FoldChange > 1. The result showed that RHBDD1 was positively correlated with a stem-like phenotype, which may be a result of CRC cell dedifferentiation via EMT.

### Positive correlation between RHBDD1 and ZEB1 from CRC cells to tumor tissues from patients

**Fig. 6:**
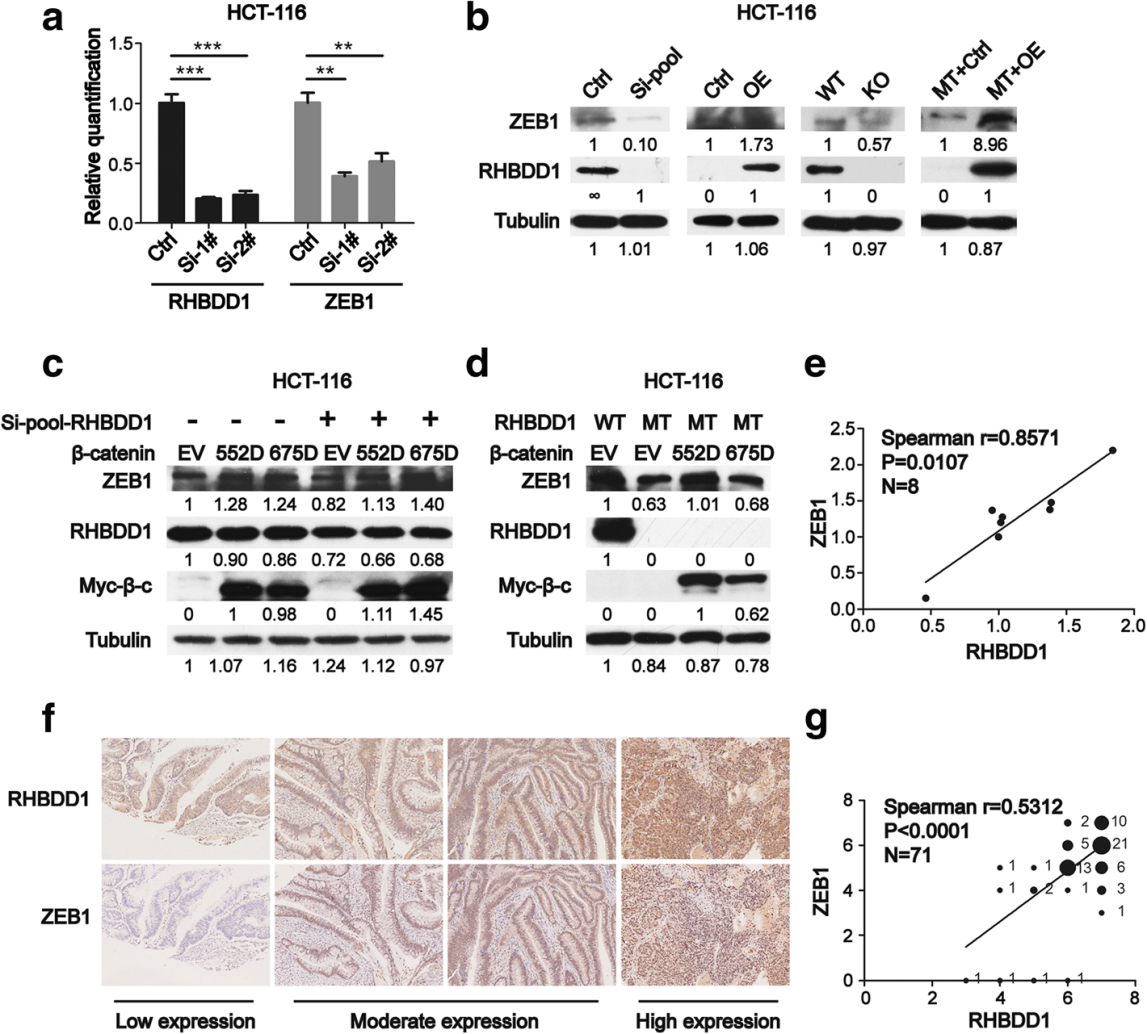
Positive correlation between RHBDD1 and ZEB1 from CRC cells to tumor tissues from patients. **a**. Q-PCR analysis of ZEB1 and RHBDD1 mRNA levels in HCT-116 cells with knockdown of RHBDD1. Ctrl, control; Si-1#, Si-RHBDD1–1#; Si-2#, Si-RHBDD1–2#; **, *P* < 0.01; ***, *P* < 0.001. **b**. Immunoblot analysis of ZEB1 and RHBDD1 protein levels when in HCT-116 cells with knocked down, over-expressed, knocked out or rescued RHBDD1 expression. Si-pool, mixture of Si-RHBDD1–1# and Si-RHBDD1–2# in a ratio of 1:1; OE, over-expression. WT, wild-type; KO, cas9 knocked out; MT + Ctrl, mutant cell line infected with lentivirus-control; MT + OE, mutant cell line infected with lentivirus-RHBDD1. **c**. HCT-116 cells were co-transfected with si-RHBDD1 miRNA or control miRNA and empty vector, S552D-β-catenin or S675D-β-catenin plasmids simultaneously, 48 h after which immunoblot analysis of ZEB1, RHBDD1 and β-catenin was performed. **d**. Wild-type HCT-116 cells and RHBDD1 mutant HCT-116 cells were transfected with empty vector, S552D-β-catenin or S675D-β-catenin constructs and then subjected to immunoblot analysis of ZEB1, RHBDD1 and β-catenin. **b, c, d**. Bands were quantified with ImageJ software. **e**. Immunoblot analysis of ZEB1 and RHBDD1 expression levels in 8 CRC tumor tissues, Tubulin was used as a loading control. Bands were quantified using ImageJ software. The correlation between ZEB1 and RHBDD1 protein levels were calculated using GraphPad Prism 5 software, the dots in the data represent the expression levels of RHBDD1 and ZEB1 relative to tubulin in the indicated tissues. **f**. Images of tissues showing the levels of ZEB1 and RHBDD1 protein expression in the tissue microarray analysis. **g**. Correlation between the levels of ZEB1 and RHBDD1 protein expression in 71 tissues of the tissue microarray analysis. The protein expression levels were evaluated using an immunostaining score as indicated in the Methods section. Numbers beside the dots represent the sample sizes with indicated immunostaining score of ZEB1 and RHBDD1. The dots’ areas are coincident with the sample sizes

We already proved that RHBDD1 can influence gene signatures downstream of the Wnt signaling pathway; thus, we further explored the exact target gene of the Wnt signaling pathway that is regulated by RHBDD1. ZEB1 is reported to be a Wnt target gene [8]. It is a potent EMT activator [27] and can regulate cell stemness [28]. Based on the results in Fig. 5, we discovered the role of ZEB1 in RHBDD1-mediated regulation of colorectal metastasis. In HCT-116 cells, Knockdown RHBDD1 can decrease (*P* < 0.01) the mRNA (Fig. 6a) level of ZEB1; knockdown or over-expression of RHBDD1 can decrease or increase, respectively, the protein (Fig. 6b) levels of ZEB1. RHBDD1 Cas9-KO HCT-116 cells showed a decreased protein level of ZEB1 relative to the level in wild-type cells (Fig. 6b). HCT-116 cells with rescued RHBDD1 expression showed recovery of ZEB1 protein expression relative to the level in control cells (Fig. 6b). We transfected the empty vector, S552D, or S675D constructs in HCT-116 cells with RHBDD1 knockdown and observed that the protein levels of ZEB1 were rescued relative to the levels in wild-type HCT-116 cells upon transfection with either S552D or S675D constructs (Fig. 6c). The same result was obtained in HCT-116 cells with stable expression of mutant RHBDD1 (Fig. 6d). These results showed that RHBDD1 can regulate ZEB1 at the protein level via p552 and p675 β-catenin.

We validated the results obtained from cell models in primary tissues. CRC tumor tissues from 8 patients were analyzed using western blotting (Additional file 2: Figure S1B). The result was scanned for gray scale, and the statistical analysis showed the protein expression levels of RHBDD1 and ZEB1 have a high degree of positive correlation (Spearman r: 0.8571, *P* = 0.0107) (Fig. 6e). Furthermore, we conducted a tissue microarray to explore the correlation between these two proteins in 71 colon cancer tissues. Fig. 6f shows that the protein expression levels of ZEB1 are coincident with RHBDD1 expression in different tissues. The statistical analysis showed that RHBDD1 and ZEB1 exhibited a moderately positive correlation in 71 colon cancer tissues (Spearman r: 0.5312, *P* < 0.0001) (Fig. 6g).

## Discussion

Since metastasis causes 90% of all cancer-related deaths in cancer patients [1], it is important to deeply understand the mechanism(s) of metastasis. Few studies have proven that rhomboid family proteins can regulate cancer metastasis. RHBDD2, a member of the rhomboid family, is reported to express a significant increase in breast primary tumors with a more disseminated disease than with less disseminated tumors [21]; overexpression in advanced stages of colorectal cancer is also observed [22]. RHBDD2 might play a role in cancer metastasis, while the mechanism is completely unknown. In this study, we proved the connection between rhomboid family protein RHBDD1 and CRC metastasis, and we also raised a possible mechanism for this effect for the first time.

**Fig. 7:**
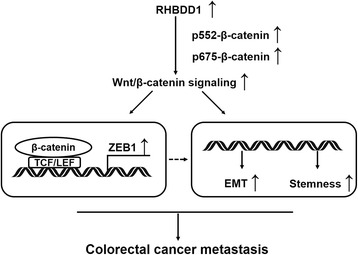
Schematic illustration of the proposed mechanism by which RHBDD1 promotes metastasis in colorectal cancer. RHBDD1 activates Wnt-signaling pathway activity mainly through increasing p552-β-catenin and p675-β-catenin to promote cell EMT and stemness. Meanwhile, RHBDD1 increases ZEB1 expression. ZEB1 is a potent EMT activator and can regulate cell stemness. Thus, EMT and stemness promote colorectal cancer metastasis

In this study, we determined that RHBDD1 is positively correlated with CRC lymphatic metastasis and distal metastasis in clinical samples, thus proving its role in cancer metastasis. The results from the cell models and animal models all showed that RHBDD1 expression can promote an aggressive phenotype in CRC cells. RHBDD1 activates Wnt-signaling pathway activity mainly through increasing p552-β-catenin and p675-β-catenin to promote cell EMT and stemness (Fig. 7). RHBDD1 increases the Wnt/β-catenin/TCF target gene ZEB1 expression (Fig. 7). We additionally proved the positive correlation between RHBDD1 and ZEB1 at the protein level in primary colon cancer samples from patients. Therefore, RHBDD1 increases Wnt signaling pathway activity, which induces increased expression of ZEB1. Then CRC cells undergo EMT and acquire stem-like phenotype, promoting tumor progression to metastasis.

The metastatic cascade can be conceptually organized into intravasation, extravasation and colonization three major phases [29]. Lymphatic metastasis is an early step of metastasis. Tumor cells dedifferentiate to regain a stem-like phenotype, which then invade blood microvessels and lymphatic vessels to transit to distant organs. RHBDD1 intensity score and the percentage of patients with positive RHBDD1 expression are significantly higher in patients with lymphatic metastasis than in patients without lymphatic disease, with *P* < 0.001, indicating RHBDD1 may have the function of promoting CRC metastasis at the intravasation stage. This phenomenon coincides with RHBDD1 promoting colorectal tumor cells undergo EMT and promoting a stem-like phenotype. RHBDD1 intensity score and the percentage of patients with positive RHBDD1 expression are significantly higher in patients with distal metastasis than in patients with local recurrence and implantation metastasis, with *P* < 0.05, indicating RHBDD1 may function of promoting CRC distal metastasis at the extravasation and colonization stages, while the correlation of RHBDD1 with distal metastasis is weaker than lymphatic metastasis. However, because the patients’ count in the cohort have records regarding the presence of local recurrence, implantation metastasis or distal metastasis was relatively small, further statistical analysis is needed.

Using RNA sequencing and dual-luciferase reporter assay, we focused on the Wnt signaling pathway. The core protein in the Wnt signaling pathway is β-catenin. GSK3β and CK1 phosphorylate ser/thr 33/37/41/45 on β-catenin, which leads to its degradation by the proteasome [30]. In the intestine, the PI3K-AKT signaling pathway phosphorylates β-catenin at ser552 to activate β-catenin-mediated TCF signaling [24, 31]. PKA phosphorylates ser552 and ser675 on β-catenin in vitro to promote activity of β-catenin-mediated TCF signaling [32]. Upon modifying RHBDD1, the total- and phosphor-β-catenin changed in a way coincident with the TOP/FOP flash reporter assay results. Additionally, we hypothesized that changes in total-β-catenin expression were key in RHBDD1 regulating CRC metastasis and that restoring total-β-catenin expression after knocking down RHBDD1 should rescue Wnt signaling pathway activity. To test this hypothesis, we stimulated HCT-116 cells with CHIR99021 after knocking down RHBDD1, and the TOP/FOP flash reporter assay results showed that the pathway activity cannot be rescued. There may be two reasons to explain this phenomenon. First, RHBDD1 regulates β-catenin downstream of GSK3α/β, and CHIR99021 cannot rescue changes in the protein levels of total-β-catenin. Second, total-β-catenin is not the key effector of RHBDD1-mediated regulation of the Wnt signaling pathway. We stimulated HCT-116 cells with CHIR99021 after knocking down RHBDD1, and conducted western blot analyses of total and phosphorylated β-catenin expression after cytosolic and nuclear isolation. The results showed that β-catenin expression in the cytosol and the nucleus were rescued, which excludes the first possibility. Therefore, changes in total-β-catenin expression were not key in RHBDD1 regulating CRC metastasis. By over-expressing the S552D and S675D constructs, the pathway activities, migration, and invasion abilities recovered in HCT-116 cells with mutant RHBDD1, proving that the Wnt signaling pathway activity is directly associated with p552- and p675-β-catenin.

The Wnt signaling pathway regulates EMT and ISCs homeostasis. In CRC, EMT can influence many types of malignant phenotypes correlated with metastasis, including the manifestation of cancer stem cells, tumor budding and drug resistance [33]. In adult intestine, Wnt/β-catenin signaling maintains the homeostasis of crypt stem cells [11]. When the pathway activity becomes dysregulated, it can induce the formation of cancer stem cells [11]. The plasticity and differentiation ability of cancer stem cells allow them to better adjust to the microenvironment via metastasis [12]. The pan-EMT and ISC signatures of HCT-116 cells showed decreases in the aggressive phenotype after knocking down RHBDD1 expression, which proved that RHBDD1 is positively correlated with the aggressive phenotype of CRC cells.

It has been reported that β-catenin and TCF4 can bind the promoter region of ZEB1 to promote its expression [8]. ZEB1 is a potent EMT activator [27] and can regulate cell stemness [28]. ZEB1 is correlated with lymphatic and distal metastasis [34] and influences metastasis via EMT in lung cancer [27]. ZEB1 also regulates tumor metastasis through the ZEB1/EMT axis in hepatoma [35], breast cancer [35, 36], and colorectal cancer [37, 38]. ZEB1 can maintain pancreatic tumor cell stemness [28]. Breast stem cells express higher levels of ZEB1 than differentiated cells [39]. ZEB1 exhibits its function through EMT and stemness the same as Wnt signaling and has been reported to be a Wnt target gene. Therefore, we predicted that RHBDD1 regulates the Wnt signaling pathway target gene ZEB1 to promote CRC metastasis. We validated the regulation of ZEB1 by RHBDD1 at the mRNA and protein level. In HCT-116 cells, knocking down or mutating RHBDD1 led to the recovery of ZEB1 protein levels in cells over-expressing either S552D-β-catenin or S675D-β-catenin. The results also showed that RHBDD1-mediated regulation of ZEB1 was directly associated with p552-β-catenin and p675-β-catenin.

The results from the cell lines were validated in tumor tissues to confirm their reliability. We proved that RHBDD1 and ZEB1 were positively correlated at the protein level by western blotting and microarray assays in tumor tissues, which will further facilitate translational medicine research on RHBDD1.

The five-year survival rate of CRC was 90.1% for patients with localized stage, while only 11.7% for patients with distant tumor spread [4]. Therefore, exploring new targeted therapy drugs are crucial for metastatic CRC therapy. RHBDD1 as an intramembrane serine protease, has the potential to become a new therapeutic target. Aberrant Wnt signaling has been associated with 90% of CRC [5], and is therefore an attractive target for therapeutic intervention. Further analyses of the molecular structure of RHBDD1 protein and its specific small molecule inhibitor are well worth discussing. Our research provides a new way of targeting Wnt signaling pathway, which functions through RHBDD1 regulating phosphor-β-catenin. We also provide a new target, RHBDD1, for clinical therapeutic research.

## Conclusions

In summary, we proved that RHBDD1 can promote CRC metastasis through the Wnt signaling pathway and ZEB1. As a membrane protein, RHBDD1 has the potential and advantage to become a new therapeutic target or clinical biomarker for metastatic CRC.

## Additional files

Additional file 1: **Table S1.** Primers and RNAi oligos used in the article. (DOCX 15 kb)

Additional file 2: **Figure S1.** A. Immunoblot analysis of the RHBDD1 levels in colorectal cancer cell lines. Tubulin was used as a loading control. B. Immunoblot analysis of ZEB1 and RHBDD1 levels in 8 colorectal cancer tumor tissues. Tubulin was used as a loading control. T, tumor tissue. Bands were quantified using ImageJ software. (TIF 3.22 mb)

## Abbreviations

CRC: Colorectal cancer
CTCs: Circulating tumor cells
EMT: Epithelial-mesenchymal transition
ERAD: Endoplasmic reticulum-associated degradation
ISCs: Intestinal stem cells
NOD/SCID: Non-obese diabetic/server combined immune-deficiency
