# Correction to: RHBDD1 promotes colorectal cancer metastasis through the Wnt signaling pathway and its downstream target ZEB1

**DOI:** 10.1186/s13046-018-0709-3

**Published:** 2018-03-16

**Authors:** Mengmeng Zhang, Fei Miao, Rong Huang, Wenjie Liu, Yuechao Zhao, Tao Jiao, Yalan Lu, Fan Wu, Xiaojuan Wang, Han Wang, Hong Zhao, Hongge Ju, Shiying Miao, Linfang Wang, Wei Song

**Affiliations:** 1 State Key Laboratory of Medical Molecular Biology, Department of Biochemistry and Molecular Biology, Institute of Basic Medical Sciences, Chinese Academy of Medical Sciences & Peking Union Medical College, Beijing, 100005 China; 2 Department of Abdominal Surgical Oncology, Cancer Hospital & Institute, Chinese Academy of Medical Sciences, Beijing, 100021 China; 3 Department of Pathology, Baotou Medical College, Baotou, 014040 China; 4 Department of Pathology, The First Affiliated Hospital of Baotou Medical College, Baotou, 014010 China

## Correction

In the publication of this article [1], there is an error in the Methods section at paragraph Cell culture and reagents and the Additional file 2: Figure S1 was erroneous linked.

The error: ‘All these cell lines were passaged fewer than 6 months after purchase for all the experiments. HCT-116, and DLD1 cells were cultured in IMDM (HyClone, SH30228.01) supplemented with 10% fetal bovine serum (Gibco, 15140–122); HCT-8 cells were cultured in RPMI-1640 medium (Cell Resource Center, IBMS, CAMS/PUMC, CCCM019) supplemented with 10% fetal bovine serum; and CACO-2 cells were cultured in MEM-EBSS medium (HyClone, SH30024.01) supplemented with 10% fetal bovine serum and nonessential amino acids (HyClone, SH30238.01).‘

Should instead read: ‘All these cell lines were passaged fewer than 6 months after purchase for all the experiments. HCT-116, and DLD1 cells were cultured in IMDM (HyClone, SH30228.01) supplemented with 10% fetal bovine serum (Gibco, 10099141); HCT-8 cells were cultured in RPMI-1640 medium (Cell Resource Center, IBMS, CAMS/PUMC, CCCM019) supplemented with 10% fetal bovine serum; and CACO-2 cells were cultured in MEM-EBSS medium (HyClone, SH30024.01) supplemented with 10% fetal bovine serum and nonessential amino acids (HyClone, SH30238.01).‘

The error: Additional file 2: Figure S1 was linking to an incorrect file.

Should instead read: Additional file 2: Figure S1 does now link to the correct Additional file 2: Figure S1.

This has now been updated in the original article [1].
