# Supplementary material for: RHBDD1 promotes colorectal cancer metastasis through the Wnt signaling pathway and its downstream target ZEB1
Source: J Exp Clin Cancer Res. 2018 Feb 9;37:22. doi: 10.1186/s13046-018-0687-5 (PMC5807852; doi:10.1186/s13046-018-0687-5)
Supplement: Supplementary file 1 — Table S1. Primers and RNAi oligos used in the article. (DOCX 15 kb) [file 13046_2018_687_MOESM1_ESM.docx]

**Additional file 1: Table S1:**

| **Primers for beta-catenin point mutant constructs:** | |
| --- | --- |
| **To obtain full-length fragments:** | |
| Forward: | GTTAAGCTTGGTACCACCACCAUGGATGGCTACTCAAGCTGATTT |
| Reverse: | CGCGGGCCCTCTAGACTCAGGTCAGTATCAAACCA |
| **To obtain point mutants fragments:** | |
| S552D forward: | ACCCAGCGCCGTACGGACATGGGTGGGACAC |
| S552D reverse: | GTGTCCCACCCATGTCCGTA CGGCGCTGGGT |
| S675D forward: | TACAAGAAACGGCTTGATGTTGAGCTGACCA |
| S675D reverse: | TGGTCAGCTCAACATCAAGCCGTTTCTTGTA |
| **RNAi oligos targeting RHBDD1:** | |
| Si-RHBDD1-1#: | GUAGAUGGUUUGCCUAUGUTT |
| Si-RHBDD1-2#: | GGAUUCUUGUUGGACUAAUTT |
| Negative control: | UUCUCCGAACGUGUCACGUTT |
| **Primers used for Q-PCR:** | |
| ZEB1 sense: | 5'-ACTCTGATTCTACACCGC-3' |
| ZEB1 anti-sense: | 5'-TGTCACATTGATAGGGCTT-3' |
| RHBDD1 sense: | 5'-ATGCAACGGAGATCAAGAGGG-3' |
| RHBDD1 anti-sense: | 5'-GCAGGAGCTATACAGTGGCTTC-3' |
| 18S rRNA sense: | 5'-ACACGGACAGGATTGACAGA-3' |
| 18S rRNA anti-sense: | 5'-GGACATCTAAGGGCATCACA-3' |
| Beta-actin sense: | 5'-GGGAAATCGTGCGTGACATTAAG-3' |
| Beta-actin anti-sense: | 5'-TGTGTTGGCGTACAGGTCTTTG-3' |
