# Supplementary figures and images for: RHBDD1 promotes colorectal cancer metastasis through the Wnt signaling pathway and its downstream target ZEB1
Source: J Exp Clin Cancer Res. 2018 Feb 9;37:22. doi: 10.1186/s13046-018-0687-5 (PMC5807852; doi:10.1186/s13046-018-0687-5)

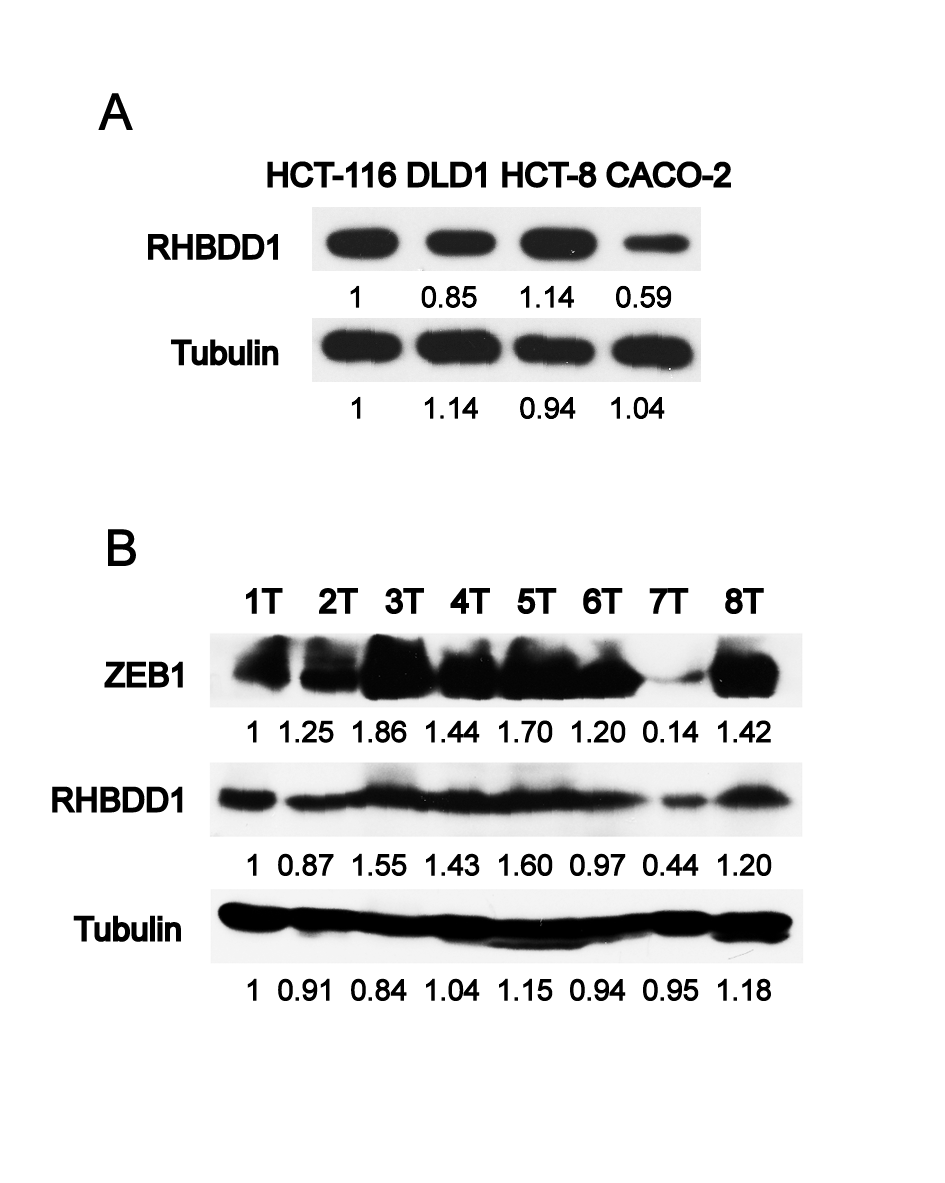

Supplement: Supplementary file 2 — Figure S1. A. Immunoblot analysis of the RHBDD1 levels in colorectal cancer cell lines. Tubulin was used as a loading control. B. Immunoblot analysis of ZEB1 and RHBDD1 levels in 8 colorectal cancer tumor tissues. Tubulin was used as a loading control. T, tumor tissue. Bands were quantified using ImageJ software. (TIF 3.22 mb) [file 13046_2018_687_MOESM2_ESM.tif]
